# Anifrolumab for Moderate and Severe Muco-Cutaneous Lupus Erythematosus: A Monocentric Experience and Review of the Current Literature

**DOI:** 10.3390/biomedicines11112904

**Published:** 2023-10-26

**Authors:** Giovanni Paolino, Giuseppe A. Ramirez, Chiara Calabrese, Luca Moroni, Vittoria Giulia Bianchi, Enrica P. Bozzolo, Santo Raffaele Mercuri, Lorenzo Dagna

**Affiliations:** 1 Unit of Dermatology, IRCCS Ospedale San Raffaele, Via Olgettina 60, 20132 Milan, Italy; 2 Unit of Immunology, Rheumatology, Allergy and Rare Diseases, IRCCS Ospedale San Raffaele, Via Olgettina 60, 20132 Milan, Italy; 3 Faculty of Medicine, Università Vita-Salute San Raffaele, Via Olgettina 58, 20132 Milan, Italy

**Keywords:** anifrolumab, lupus, cutaneous, discoid, CLA-IGA

## Abstract

Refractory cutaneous manifestations constitute a significant unmet need in patients with cutaneous lupus (CLE), even in the setting of systemic lupus erythematosus (SLE) with otherwise good control of inflammatory manifestations. Anifrolumab, an anti-interferon I receptor monoclonal antibody has recently been approved for serologically positive SLE with or without CLE, but real-life efficacy and safety data are currently limited. In addition, relatively limited evidence exists about the spectrum of cutaneous manifestations potentially benefitting from anifrolumab treatment and about the optimal clinimetrics to monitor treatment efficacy. While summarising current evidence on the topic in the literature, we report on four patients with SLE and refractory CLE who were successfully treated with anifrolumab. We also describe the potential usefulness and complementarity of the cutaneous lupus activity investigator’s global assessment (CLA-IGA) in assessing cutaneous activity in patients treated with anifrolumab.

## 1. Introduction

Cutaneous lupus erythematosus (CLE) is an autoimmune and chronic disease characterised by a wide range of cutaneous and systemic clinical manifestations. CLE is generally divided into different subgroups, consisting of acute CLE, subacute CLE, chronic CLE, and intermittent CLE. Up to 80% of patients with systemic lupus erythematosus (SLE) may develop muco-cutaneous lesions, and about 25% of SLE patients have muco-cutaneous involvement at time of diagnosis. Unfortunately, CLE and discoid lupus erythematosus (DLE) may have an important impact in the daily life of patients, since cutaneous lesions may induce atrophy, scars and dyspigmentation [1,2,3,4,5,6,7,8,9,10,11].

In order to monitor CLE progression and the responses to treatments, two main algorithms have been validated: the Cutaneous Lupus Erythematosus Disease Area and Severity Index (CLASI) and the revised CLASI (RCLASI) index. The CLASI algorithm generates two scores, CLASI-activity (CLASI-A) and damage (CLASI-D). Recently, a new pragmatic score, the cutaneous lupus activity investigator’s global assessment (CLA-IGA), has been developed and rapidly included in clinical trial assessments for treatment efficacy [12].

CLE often constitutes a challenge for clinicians and finding the best treatment for patients is not always simple. The first-line treatment of CLE is based on short courses of topical steroids in order to avoid secondary side effects, such as atrophy and telangiectasia. The most used topical steroids are hydrocortisone 0.5% (for facial lesions and areas with thin skin), while clobetasol cream and mometasone furoate cream are steroids with higher potency employed for other cutaneous areas (e.g., trunk and limbs). Topical calcineurin inhibitors (such as 0.03% or 0.1% tacrolimus or 1% pimecrolimus) can be used as second-line treatments with lower side effects in the long period and are useful above all for facial cutaneous lesions. Finally, topical retinoids (such as tretinoin 0.5% cream) can be useful in hyperkeratotic CLE and/or in cutaneous lesions not responding to topical steroids and calcineurin-inhibitors [5].

Regarding systemic treatments, first-line therapies include antimalarial agents (such as hydroxychloroquine [HCQ], chloroquine [CQ] and quinacrine [Q]). Systemic corticosteroids are recommended as a first-line treatment in highly active and/or severe CLE. Finally, second-line therapies include immunosuppressive and immunomodulatory agents (such as methotrexate, MTX, mycophenolate mofetil, MMF, azathioprine, AZA, cyclosporine A, CsA, cyclophosphamide, CYC, retinoids, dapsone, thalidomide, THL, lenalidomide, and iberdomide) and B-cell targeted biologic therapies (such as rituximab, RTX and belimumab, BEL). Potential new treatments encompass Janus kinase inhibitors (ruxolitinib or baricitinib), as well as anti IL-12 and anti IL-23, such as ustekinumab. Anifrolumab, a human monoclonal antibody binding type I interferon receptor, has been recently approved by EMA and FDA for the treatment of SLE [1,2,3]. By targeting type I interferon signalling, anifrolumab tackles a key factor in the pathogenesis of SLE and CLE [13,14]. In fact, dysregulated interferon alpha-driven responses resembling antiviral inflammation constitute a hallmark of lupus and account for facilitated maturation of myeloid-derived dendritic cells presenting autoantigens. This mechanism, in turn, favours downstream T- and B-cell activation towards autoimmunity [15,16,17]. Notably, natural anti-interferon antibodies associate with milder SLE and enhanced susceptibility to viral infections [18]. Consistent with the evolutionary role of interferon in the first-line control of infections, interferon responsiveness (type I and type III) is particularly pronounced in the skin even under physiological conditions [19]. In addition, the skin of patients with SLE has enhanced susceptibility to cell damage by exogenous noxae, along with interferon hyperresponsiveness to inflammatory stimuli, thus constituting a major site of autoantigen sensitisation and triggering of systemic inflammation [20,21].

Here, we report on our preliminary experience of CLE patients treated with anifrolumab, evaluating their response according to the CLASI, SLE disease activity index (SLEDAI) scores, and to the novel CLA-IGA score. We also provide a comprehensive review of published literature on anifrolumab and CLE so far.

## 2. Case Series

Starting from mid-2022, an early access programme for the “compassionate” use of anifrolumab was available in Italy before the drug was definitively available on the market and reimbursable by the National Health System. In the framework of this programme, we treated four women with SLE and refractory cutaneous involvement with intravenous 300 mg anifrolumab every 4 weeks. Upon informed consent, the patients were also enrolled in a long-term observational study (Pan-Immuno Research Protocol), conforming to the Declaration of Helsinki, and approved by the Institutional Review Board at San Raffaele Hospital, Milan, Italy. Here, we describe their clinical characteristics before and after treatment with anifrolumab along with a review of the literature. Data collection encompassed demographics, SLE history including clinical manifestations, and treatments along with clinical and laboratory features at enrolment and during follow-up. Patient-skin phototype was classified according to the Fitzpatrick’s method [22,23]. General lupus clinimetrics included the SLEDAI 2000 (SLEDAI-2K), while CLASI and CLA-IGA served as skin-specific scores. SLEDAI-2K and CLASI were calculated with an in-house software, developed for SLE patient monitoring and assessment [24].

### 2.1. Case 1

**Figure 1:**
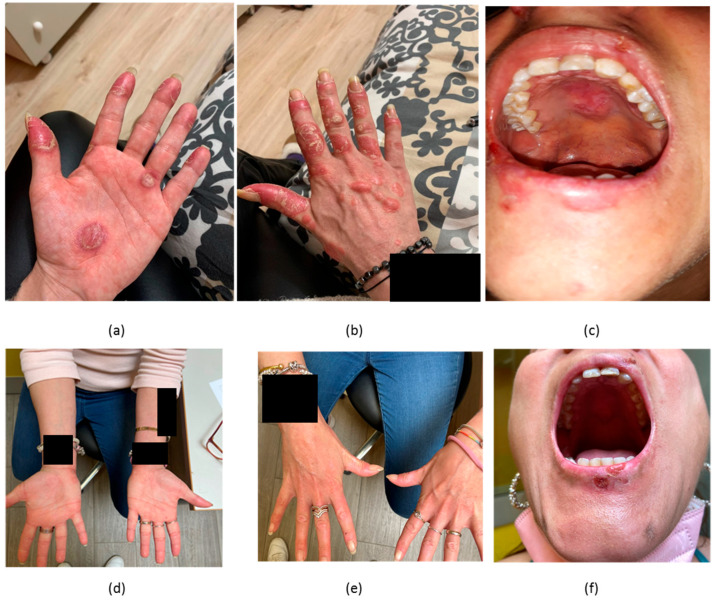
(**a**) cutaneous lesions in the palms in patient #1 with CLE before treatment; (**b**) cutaneous lesions in the back of the hands in the same patient with CLE before treatment; (**c**) lesions in the hard palate in a patient with CLE before treatment; (**d**) resolution of lesions in (**a**) assessed at 18 weeks of therapy with anifrolumab; (**e**) resolution of lesions in (**b**) assessed at 18 weeks of treatments with anifrolumab; (**f**) improvement of lesions in the hard palate (**c**) assessed at 18 weeks of treatment with anifrolumab.

Patient #1 was a 37-year-old woman, phototype III, with a four-year history of SLE with joint and skin manifestations along with positive anti-nuclear antibodies (ANA), anti-DNA antibodies (ADNA) and low complement. Cutaneous manifestations encompassed discoid lupus, SCLE, malar rash, alopecia, oral and genital ulcers and had been refractory to antimalarials, corticosteroids, MMF, MTX, CsA, AZA, BEL, baricitinib and evobrutinib. She was then started on anifrolumab on a background of MMF, corticosteroids and HCQ. At baseline, she had CLASI-A = 27, CLASI-D = 6, CLA-IGA = 4, SLEDAI-2K = 14. In terms of serology, she had low-titre positive ADNA, low C3 (0.86 g/L, normal range 0.9–1.8), normal C4 (0.16 g/L, normal range 0.1–0.4). She experienced a rapid clinical improvement starting from the first month of anifrolumab treatment (Figure 1). After four months, she had CLASI-A = 3, CLASI-D = 6, CLA-IGA = 2, SLEDAI-2K = 6 and was able to reduce her prednisone-equivalent dose from 10 mg/day to 3 mg/day. Although her ADNA levels decreased below the laboratory positive threshold, complement levels remained low (C3 = 0.79 g/L, C4 = 0.1 g/L). Over the course of 23 weeks of treatment, she had one episode of herpes labialis and one of vaginal candidiasis.

### 2.2. Case 2

**Figure 2:**
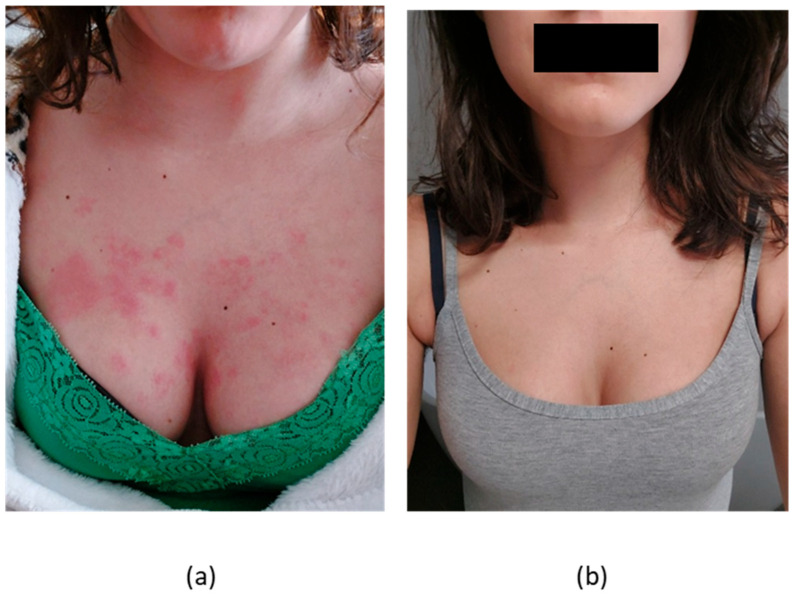
(**a**) cutaneous lesions in patient #2 with CLE before treatment; (**b**) a complete resolution documented at 38 weeks of treatment.

Patient #2 was a 25-year-old woman, phototype III, with a twelve-year history of SLE with class IV lupus nephritis, arthritis, skin manifestations, myocarditis, positive ANA, ADNA, anticardiolipin antibodies and low complement. Her systemic manifestations had been responsive to CYC and, more recently, MMF. However, while on MMF, she had persistent steroid dependency to control her skin manifestations, consisting of photosensitive rashes (Figure 2) and chronic urticaria/angioedema. She was then started on anifrolumab with abatement of CLASI-A from twelve to zero after the first month of treatment, which was maintained after 46 weeks of treatment along with SLEDAI-2K reduction from four to two and persistently negative CLASI-D. CLA-IGA was 3 at baseline and 0 after four months. Serologically, she had normal C3 (1.19 g/L) and C4 levels (0.13 g/L) and negative ADNA at treatment start and slightly lower C4 levels (0.09 g/L) at last observation, along with stable C3 (1.27 g/L) and ADNA levels. During treatment, she had recurrent vaginal candidiasis, which resolved with MMF temporary discontinuation and appropriate systemic antifungal treatment.

### 2.3. Case 3

**Figure 3:**
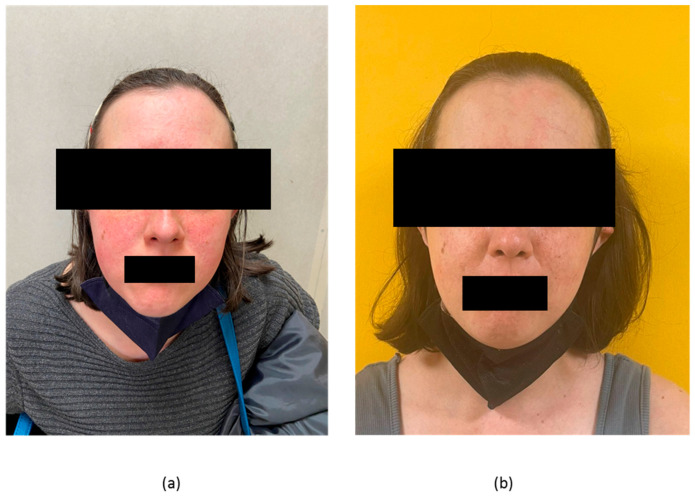
(**a**) cutaneous lesions in patient #3 with CLE at week 10 of treatment; (**b**) improvement documented at 16 weeks of treatment.

Patient #3 was a 36-year-old woman, phototype II, with a history of class IV lupus nephritis complicated by posterior reversible encephalopathy syndrome thirteen years before. Her SLE historical manifestations also included pancytopenia, arthritis, along with skin manifestations (malar rash, alopecia, DLE, cutaneous vasculitis). She was positive for ANA, ADNA, anti-Sm, anti-Ro, anti-RNP antibodies, lupus anticoagulant and she had low complement. Although her non-cutaneous manifestations had responded to CYC, MMF and B-cell depleting agents, her malar rash and photosensitivity persisted despite medium-dose steroid treatments. She was started on anifrolumab on a background of MMF, corticosteroids (HCQ had been recently discontinued for suspect retinal toxicity) and topical pimecrolimus. At that time, she had medium-titre positive ADNA and low C3 (0.74 g/L) along with normal C4 levels (0.16 g/L). She had a modest response when assessed at 10 weeks of treatment but showed almost complete lesion resolution after the fourth infusion (Figure 3). Accordingly, her CLASI-A dropped from five to one from baseline to week 16. CLA-IGA was 2 at baseline and 1 after 16 weeks. Her SLEDAI-2K remained stable at six points, due to concomitant persistence of medium-titre positive ADNA and low C3 (0.8 g/L) with normal C4 (0.18 g/L). She had no adverse events correlated to anifrolumab administration.

### 2.4. Case 4

**Figure 4:**
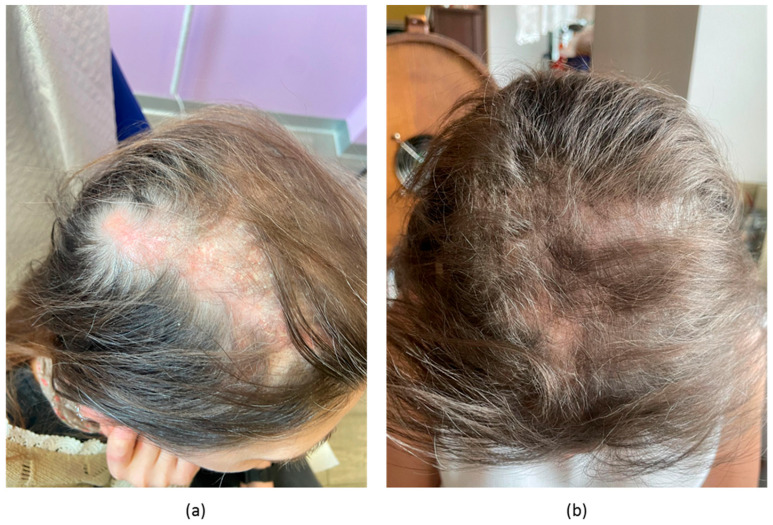
(**a**) scalp alopecia in patient #4 before treatment; (**b**) partial improvement documented after 8 weeks of treatment.

Patient #4 was a 44-year-old woman, phototype II, with a nine-year history of SLE with previous renal, haematological, and musculoskeletal involvement along with positive ANA, ADNA, anti-phospholipid antibodies and low complement, but recently dominated by mucocutaneous manifestations, encompassing alopecia, chilblain lupus, digital vasculitis, Raynaud’s phenomenon, oral ulcers and photosensitivity. These manifestations were refractory to MMF and BEL in combination with hydroxychloroquine and corticosteroids. In addition, corticosteroid doses could not be raised and maintained significantly for concomitant osteoporosis. The patient was then started on anifrolumab. Her pre-treatment CLASI-A was 17, while CLA-IGA was 3. She had a baseline SLEDAI-2K of 11 points, which included low C3 (0.77 g/L) and C4 (0.09 g/L) along with medium-titre ADNA. After eight weeks of treatment, she had partial, but significant improvement in cutaneous activity (CLASI-A = 9 points, CLA-IGA 1; Figure 4). Almost complete local and systemic inflammation resolution was observed at week 12 after treatment (CLASI-A 2, SLEDAI-2K 4). Complement C3 (0.96 g/L) and C4 levels (0.12 g/L) normalised while ADNA remained stably positive. Her CLASI-D damage increased from eight to 13 points. She had no significant adverse events associated with anifrolumab administration.

## 3. Summary of Clinical Data and Review of Literature

Globally, all patients in our series had less than 50 years of age (range 25–44) despite a median disease duration of nine years (range 5–12). All patients had muco-cutaneous involvement with main localisation in the head/neck region (*n* = 3), trunk (*n* = 4), limbs (*n* = 3) oral (*n* = 2) and genital areas (*n* = 1). Three patients had a history of lupus nephritis and one had DLE. All patients received anifrolumab in addition to MMF and corticosteroids and were able to taper corticosteroids to low doses or to discontinuation. Regarding topical treatments, pimecrolimus was used by one patient. No serious adverse reactions were observed. Accordingly, no patient discontinued anifrolumab. In terms of cutaneous clinimetrics, we observed a median CLASI-A decrease of 7.5 points (range 0–20, *n* = 4) at week 4 and of 12 points (range 4–24) at week 16. Baseline CLA-IGA was 3 in two patients, one patient had CLA-IGA = 2 and one CLA-IGA = 4 at time of anifrolumab start. CLA-IGA decreased in all patients within eight weeks and was negative at the 38-week assessment in patient #2 who was the longest treated patient. Variations in C3, C4 and ADNA levels before and after treatment were not significant (*p* = NS for all three parameters by Wilcoxon signed-rank test). Patient clinical characteristics and course of treatment are summarised and compared with those of subjects treated with anifrolumab so far reported in the literature in Table 1.

To date, nine reports on the response to anifrolumab therapy in SLE patients with muco-cutaneous lesions have been published in the literature [2,3,5,6,7,8,9,10,11]. In total 46 patients have been described, with a mean age of 38 years (SD = 9.3; ranging between 19 years and 75 years. (Table 1). All patients were female, with phototype I-IV in most cases (21/46) [22]. DLE (29/46), followed by CCLE (3/46) were the most frequent CLE subtypes. The mean duration of disease was 13.2 years (ranging between 3 and 26 years). The mean CLASI-A at the time of treatment start was 19.1 (ranging between 15 and 26). All reported patients had a 50% or more reduction in cutaneous disease activity within 16 weeks. Except for three patients (3/46), anifrolumab has been associated to other therapies: hydroxychloroquine has been the most frequently associated treatment (26/46), followed by steroids in 10/36 patients. Mucosal involvement has been reported in 11/46 patients, and consistently had a good response to the treatment in all studies. Side effects have been detected in only 10/46 patients, with viral reactivation in four patients and candidiasis in only one patient. Among them only one patient discontinued anifrolumab due to herpes zoster oticus with unilateral high-frequency sensorineural hearing loss [3].

## 4. Discussion

In this short case series, we reported on four consecutive patients with SLE and challenging cutaneous manifestations who were treated with anifrolumab. The clinical features of our patients were similar to those of patients reported in the literature both in terms of demographics, clinical features at treatment start and during disease history, treatment history. Similar to previous reports, we observed a significant improvement in cutaneous manifestations, along with concordant reduction in CLASI-A and SLEDAI-2K, when reported. Consistent with larger studies, we did not observe significant variations in SLE serological parameters (complement levels, ADNA) [4]. Here, we also show that CLA-IGA might be a simple and useful score to assess anifrolumab responses in clinical practice [12].

Anifrolumab has recently been approved by regulatory authorities for use in patients with antinuclear antibody-positive SLE and moderate to high disease activity, following the encouraging results of two phase III clinical trial studies (TULIP-1 and TULIP-2) [25,26]. In the TULIP-2 trial, anifrolumab not only met its primary end point, consisting of a significantly higher reduction in SLE activity measured through the British Isles Lupus Assessment Group-based composite lupus assessment (BICLA) score compared to the standard of care, but it also demonstrated benefits in lupus skin manifestations. In fact, while 49% of patients with moderate to high skin activity (baseline CLASI-A at least 10) receiving anifrolumab achieved a reduction of 50% or more on the CLASI score, only 25% of patients with comparable cutaneous activity who received the standard of care were able to achieve the same result [4]. Consistent with this evidence, real-life observational studies, including ours, support a prominent efficacy of the drug in the mucocutaneous domain of lupus pathophysiology.

A multitude of factors may account for anifrolumab efficacy in CLE. Lupus skin is particularly prone to type I interferon signalling since: (1) para-physiological exposure to environmental stimuli (such as ultraviolet rays) prompts constant release of cell death debris and nucleic acids activating antiviral-like responses; (2) keratinocytes are physiologically endowed with tonic release of interferon kappa (another type I interferon), which primes resident and tissue-infiltrating cells to interferon alfa, beta and omega stimulation, and this feature is constitutionally enhanced in SLE; (3) interferon lambda (type III interferon) is selectively expressed in the skin and synergises with type I interferon signalling; (4) type I interferon-secreting plasmacytoid dendritic cells are preferentially recruited in damaged skin compared to other tissues [19,20,21]. Notably, despite this encouraging mechanistic and clinical evidence, anifrolumab is only approved for patients diagnosed with SLE, which excludes patients with skin-limited lupus. Nonetheless, CLE-specific trials are planned to begin to address this issue (NCT06015737).

Safety data from registration trials and real-life observational studies (including this series) suggest a potential association between the use of anifrolumab and the development of mild infections. In our series, on-treatment infections were easily controlled by antimicrobial treatments and possibly by the de-escalation of background immunosuppression, which globally supports anifrolumab use in a wide target population. Consistently, long-term data from registration trials do not show a rise in severe infections with the use of anifrolumab. Interestingly, these data also suggest that severe acute respiratory syndrome coronavirus 2 (SARS-CoV-2) clinical responsiveness, which is highly dependent on type I interferon signalling [27], is not significantly affected by anifrolumab, at least in vaccinated subjects [28].

Rapid induction of clinical improvement constitutes one of the main strengths of anifrolumab treatment. This aspect is very important in cutaneous lesions in order to reduce the development of scarring lesions, dyspigmentation and scarring alopecia that may have an impact in the daily life of the patients. Indeed, according to the literature and to our experience, anifrolumab also prompts valid responses in patients with DLE, which is known to cause permanent skin damage. Furthermore, mucosal manifestations might also be effectively targeted by anifrolumab adding to the spectrum of challenging lupus features potentially targetable by the introduction of this drug in the therapeutic weaponry [8,9]. Finally, the low rate of side effects makes this treatment highly manageable and potentially associated with high-patient compliance.

## 5. Conclusions

Growing real-life evidence corroborated by data from a series of consecutive patients with SLE and refractory CLE supports the use of anifrolumab as a rapid, effective treatment for lupus skin manifestations and confirms its relatively good safety profile. Further studies are necessary to identify other potential areas of selective anifrolumab efficacy and possibly extend its indication to patients with skin-limited lupus besides patients with SLE. In this context, the use of domain-specific clinimetrics including CLA-IGA might have a non-redundant role in accurately measure treatment response.

## Figures and Tables

**Table 1:** Real-life evidence of anifrolumab efficacy and safety for CLE so far.

Ref.	*n*	Age, Gender	Phototype	Body Area	CLE Subtypes	Mucosal Involvement	Disease Duration (years)	CLASI-A	SLEDAI	Concomitant Therapies	Side Effects	Response
Khan et al. [5]	2	39, F	II	Chest	SCLE	No	18	NR	NR	PDN	No	Yes
		28, F	II	Hands	CCLE	No	NR	NR	NR	PDN	No	Yes
Chasset et al. [7]	11	35	I–IV (7) V–VI (4)	NR	DLE (10), SCLE (4), Chilblain lupus (1),	Yes (4)	12 (3–23)	15 (4–35)	8 (4–22)	HCQ (10) CQ (1) MTX (2) MM (2) Thalidomide (2) Steroids (9)	COVID-19 (1), herpes zoster (1), palmar warts (1), mucosal candidiasis (1)	Yes
Kowalski et al. [6]	6	48 (37–66)	I–III (3) V–VI (3)	Trunk, limbs, head/neck	DLE (4) CCLE (2)	No	10.5 (2–26)	NR	NR	HCQ (3), AZA (1), steroids (2), CQ (1)	VZV (1)	Yes
Pluss et al. [10]	1	30, F	II	Chest and upper limbs	SCLE	No	15	17	10	MM	No	Yes
Trentin et al. [11]	2	31 59	III III	Head/Neck Head/Neck with scarring alopecia	DLE DLE, chilblain	No No	NR NR	26 24	13 10	MTX + steroids No	No	Yes Yes
											No	
Shaw et al. [9]	8	42.5 (19–75)	I–III (2) IV–V (3) VI (3)	NR	DLE	NR	NR	17.1	NR	Steroids (1), HCQ (4), MMF (2), IVIG (2), THL (1), ACT (1), None (2)	None	Yes
Shaw et al. [8]	7	28 (30–40)	IV (3) V (4)	Lips (6), Hard palate (6), Tongue (1), nasal mucosa (1)	SLE + DLE	Yes	NR	NR	8 (4–12)	HCQ (6), MMF (3), ACT (1), THL (1), PDN (1) None	No	CR 86% PR 14%
Blum et al. [2]	3	35 (22–51)	VI (3)	Head/neck (3), limbs (1)	SLE + CLE	No	11.3 (9–13)	NR	NR	HCQ (3), MM (2), AZA (1)	No	Yes
Carter et al. [3]	7	46 (33–64)	NR	Head/neck, trunk, limbs (3)	DLE (5), chilblain lupus (1), SCLE (1)	NR	17 (6–26)	17	NR	PDN (3)	UTI (2), URI/bronchitis (1) and otitis externa (1). COVID-19 + polydermatomal shingles and VZV oticus (1)	Yes ^1^
Our case series	4	35.5 (25–44)	II 2 III 2	Trunk (4), head/neck region (4), hands (1), scarring alopecia (1), oral (2), genital (1)	SLE with muco-cutaneous involvement (3), DLE (1)	Yes	9 (5 years–12 years)	19.5 (12–27)	10.25	HCQ (4), MMF (4), PDN (3)	Genital candidiasis (2), HSV1 (1)	Yes

ACT: acitretinoin; AZA: azathioprine; CQ: chloroquine; CR: complete remission; DLE: discoid lupus erythematosus; HCQ: hydroxychloroquine; HSV: herpes simplex virus; IVIG: intravenous immunoglobulins; MMF: mycophenolate mofetil; NR: not reported; PDN: prednisone; PR: partial remission; THL: thalidomide; URI: upper respiratory tract infection; UTI: urinary tract infection; ^1^: one patient discontinued therapy due to serious adverse reaction.

## Data Availability

The data are available upon request to the corresponding author.
